# Relationships between somatic anxiety, cognitive anxiety, self-efficacy, and emotional intelligence levels in university physical education students

**DOI:** 10.3389/fpsyg.2022.1059432

**Published:** 2023-01-12

**Authors:** Isabel Mercader-Rubio, Nieves Gutiérrez Ángel, Sofia Silva, Ana Moisão, Sónia Brito-Costa

**Affiliations:** ^1^Departamento de Psicología, Universidad de Almería, Almería, Spain; ^2^Polytechnic of Coimbra, Coimbra Education School, Research Group in Social and Human Sciences (NICSH), Coimbra, Portugal; ^3^Centro de Estudos Interdisciplinares (CEIS 20), Universidade de Coimbra, Coimbra, Portugal; ^4^Centro de Investigação em Educação de Adultos e Intervenção Comunitária (CEAD), Coimbra, Portugal; ^5^Polytechnic of Coimbra, Institute of Applied Research (i2A), Coimbra, Portugal; ^6^Polytechnic of Coimbra, Human Potential Development Center (CDPH), Coimbra, Portugal

**Keywords:** somatic anxiety, cognitive anxiety, emotional intelligence, university students, physical education, self-concept

## Abstract

One of the most studied topics nowadays, from psychology in general, and from sport psychology, is anxiety. In fact, research on anxiety has been approached from various theoretical perspectives ranging from psychoanalysis, behaviorism, or more recently, those theories that take into consideration the importance of affective, rational, and emotional processes. The aim of this study is to analyze the levels of anxiety and emotional intelligence, and their relationship. The sample is composed of 165 university physical education students with a mean age of 20.33 years (SD = 3.44), (70.9% male and 27.9% female). We used the CSAI-2 questionnaire (to measure cognitive anxiety, somatic anxiety, and self-confidence), and the TMMS-24 (to measure emotional intelligence). The main findings of this research highlighted the presence of significant correlations between emotional clarity and emotional regulation, self-confidence, cognitive anxiety, and somatic anxiety. Therefore, we conclude that sporting performance is influenced by various variables of different kinds, including emotions, and highlight the importance of the incorporation of the emotional component in the field of sport.

## 1. Introduction

When the word anxiety is mentioned, it refers to a state that produces a feeling of insecurity that can act on both physical and psychological levels and that, therefore, can generate symptoms such as feelings of unease, diffuse fears, insecurity, or motor reactions such as tremors, muscle pain, nervous tics, among various others (Botelho Vinhais and Salselas, 2013; Sarudiansky, 2013; Sepúlveda-Páez et al., 2019; Aguinaga et al., 2021; Moisao et al., 2022). Therefore, anxiety is a variety of stress answers to a situation perceived as aversive that activates avoidance mechanisms and is characterized by concern and fear about the possibility of harm, whether physical or psychological, which is accompanied by an increase in physiological activation because of the threat assessment (Pineda-Espejel et al., 2019). This construct is understood as an emotion that appears in response to the way competition is interpreted and evaluated (Weinberg and Gould, 2010) and causes negative reactions, worry, and thoughts that can alter attentional processes and other cognitive functions (Silva et al., 2015; Pulido et al., 2021). This is one of the psychological variables that we are going to study, and for this reason, it is necessary to explain and describe its role in the sporting environment.

There is no doubt that in sporting life, there are numerous factors that affect the perception of threat such as the place where the competition takes place, the athlete-coach relationship, the performance level of the opponent, the expectations placed on the competition, etc. (Granero-Gallegos et al., 2011; Botelho Vinhais and Salselas, 2013; Kumar, 2016; Vaca García et al., 2017; Torrealva and Bossio, 2018; Pineda-Espejel et al., 2019; Sepúlveda-Páez et al., 2019). In particular, when faced with demands that can be interpreted as threatening, it is crucial to understand the sport competition at the beginning and during the competitive process to understand the imbalance between the demand and the response perceived by the athlete (Prieto et al., 2015; Baro et al., 2016; Jara-Moreno et al., 2020; Marholz et al., 2022), and to the judgment made both personally and by the immediate environment about their ability to face the competition and the result they may obtain (Gaetano et al., 2015; Gómez et al., 2016; Del Bosque, 2019; Mayorga et al., 2020). In short, competition corresponds to a situation that causes uncertainty about the results of the competition, so the greater the expectation of those results, the greater the threat of failure and the greater the feeling of anxiety (Guillén and Álvarez-Malé, 2010; Ries et al., 2012; Liberal et al., 2014; Hofrege et al., 2018; Medeiros et al., 2021).

In this sense, the specific literature on sport psychology focused on assessing the anxiety of athletes refers that one of the most widely used inventories to measure anxiety-trait-state has been the State-Trait Anxiety Inventory (STAI) by Spielberger et al. (1970). Differentiating this duality, two are distinguished (Ramis et al., 2013): one to assess trait anxiety, namely the Sport Competition Anxiety Test (SCAT – Martens and Schwenkmezger, 1979) and other to assess state anxiety, namely the Competitive State Anxiety Inventory (CSAI – Martens and Schwenkmezger, 1979). However, anxiety should also be assessed for its cognitive and somatic dimensions (Del Bosque et al., 2022). Therefore, we are faced with a dual conceptualization of the psychological construct of anxiety: cognitive anxiety and somatic anxiety. Thus, if we refer to cognitive anxiety, we are referring to those negative expectations that we impose on our thinking and that significantly affect sports performance. On the other hand, if we refer to the unpleasant sensations that the body suffers such as tachycardia, trembling, and difficulty in breathing, among other physiologic alterations, we are referring to somatic anxiety (Martens et al., 1990; Haase, 2021; Vivar-Bravo et al., 2022; Brito-Costa et al., 2022).

The analysis of anxiety in the field of sports corresponds to a research topic that is currently the subject of a large amount of research (López et al., 2020). In this regard, from the review of the specific literature on this topic, three well-defined lines of these two dimensions stand out: The first line would be the empirical evidence indicating that they are caused by different types of antecedents. In the cognitive dimension, it is the expectation of success and in the somatic dimension, it would intervene in the context prior to the competition (Mellalieu et al., 2009; Ries et al., 2012). The second line is the conceptual autonomy of both dimensions, not only in terms of the duration time of both but also in the effects they have on performance. The third line is the demonstration of the different methods used to reduce anxiety levels in athletes (Granero-Gallegos et al., 2017; López et al., 2020; Cahuasqui, 2022; Morales-Beltrán et al., 2022).

All these issues resulted in the reformulation of the instruments for measuring sport anxiety, specifically the CSAI to take into account the anxiety response, and the two dimensions mentioned, cognitive and somatic, addressing the subscale of self-confidence understood as the degree of certainty that athletes and people, in general, have about their ability to succeed in a task and above all to trust in achieving their purposes (Fernández et al., 2007; Ramis et al., 2010; Fernandes et al., 2012; López Walle et al., 2014).

Another psychological variable analyzed in this study is emotional intelligence, currently considered a crucial psychological factor by Balk et al. (2017) and essential to incorporate in the intervention programmers carried out by the sports psychologist, as it is currently considered a determining factor in the levels of anxiety of the athlete (Zurita et al., 2018; Castro-Sánchez et al., 2020), and even with the level of life satisfaction (Barwick et al., 2022).

We are committed to the skill model of emotional intelligence (Mayer and Salovey, 1997) which understands it as a set of skills (Fernández-Berrocal and Extremera, 2008; Mayer et al., 2008), with a dual intention: the adaptability of the subject to the context, and the regulation of their emotions (Cabello et al., 2010). Therefore, it is a model made up of different components, each, in turn, made up of different skills that develop in different directions and are developed according to their complexity (Salovey et al., 2008). These are: the refocused regulation of emotions, the judgment and study of emotions, the emotional facilitation of thought, and the clairvoyance, appreciation, and expression of emotions (Fernández-Berrocal and Extremera, 2005; Cabello et al., 2006; Extremera and Fernández-Berrocal, 2009; García del Castillo-López et al., 2013).

In this sense, the purpose of our study arises from the need to investigate the link between these two psychological variables, for which we posed the following research questions: Can emotional intelligence be a protective factor for anxiety? That is, if we work on the ability to regulate, analyze, think, and express their own emotions and those of others, will we also be teaching the athlete to control symptoms of anxiety such as discomfort, fear, insecurity, or other negative reactions, worries and thoughts that can alter attentional processes and other cognitive functions?

Bringing together all these contributions and questions, the general objective of this study is to examine the levels of emotional intelligence and the levels of anxiety in a sample of university students of physical education, as well as the type of relationship established between both psychological variables. As specific objectives, we propose to analyze the levels of each of the dimensions of emotional intelligence: attention, clarity, and emotional regulation and their influence on anxiety. As well as to analyze each of the types of anxiety: Somatic and cognitive, and their relationship with emotional intelligence. Finally, we aim to investigate the influence that self-efficacy has on emotional intelligence and what kind of relationship exists between both psychology variables.

To align the research questions with the variables under study, we established the following hypotheses:

*Hypothesis 1*: There is a direct and positive relationship between emotional intelligence and self-confidence.

*Hypothesis 2*: Athletes with higher levels of emotional attention have lower levels of cognitive anxiety and somatic anxiety.

*Hypothesis 3*: Athletes with higher levels of emotional clarity have lower levels of cognitive anxiety and somatic anxiety.

*Hypothesis 4*: Athletes with higher levels of emotional regulation have lower levels of cognitive anxiety and somatic anxiety.

## 2. Materials and methods

### 2.1. Participants

This study uses a descriptive, quantitative and cross-sectional methodology, based on an *ex post facto*, retrospective, and comparative design, in order to analyze the data from a sample comprising a total of 165 physical education students from a public university, studying degrees related to physical activity and sport sciences (both undergraduate and master’s degrees), with an average age of 20.33 years, with a standard deviation (SD = 3.44). 70.9% (*N* = 117) were men and 27.9% (*N* = 46) were women.

The sample size was calculated using the Soper *a-priori* sample size calculator for structural equation models (Soper, 2022). Thus, based on six observable variables, one latent variable, with an anticipated effect size of 0.30, probability level of 0.05, and desired statistical power level of 0.95, the minimum recommended effect size was 200 cases, meaning the number of participants in our study was close enough to the population size suggested. The type of sampling used corresponds to simple random sampling, since the participants in the study correspond to the students who attended class on the specific day on which the questionnaires were administered and who, after having been informed and given their consent, decided to participate in the study. The inclusion criteria were that the sample was made up of students enrolled in the 2021/2022 academic year in any of the 4 years of the Bachelor’s or Master’s degree in Physical Activity and Sport Sciences. All participants had to express their consent to participate in this study by signing it expressly (official model of the University of Almeria) and that they were of legal age. It should be noted that of the total sample collected (*N* = 173), 6 incomplete questionnaires were initially excluded. A further 2 were finally eliminated due to a high level of acquiescence bias in the responses.

### 2.2. Instruments

To measure emotional intelligence, we used the TMMS-24 Spanish version (Fernandez-Berrocal et al., 2004). This is a self-report instrument that measures the different dimensions of emotional intelligence (8 items for each dimension and a total of 24 items as a whole) using a Likert-type scale (1–5). The psychometric properties are appropriate in terms of reliability (Cronbach’s alpha) (attention: *α* = 0.92; clarity: *α* = 0.84; repair: *α* = 0.84) (Górriz et al., 2021). In the present study, we obtained Cronbach’s alpha scores (*α* = 0.84).

To measure anxiety, we used the CSAI-2 Spanish version (Martens et al., 1990; Capdevila, 1997), subsequently revised and used by other researchers (Arruza et al., 2001; Telletxea, 2008), which assesses somatic anxiety, cognitive anxiety, and self-confidence, composed entirely of 27 items on a Likert-type scale (1–4). The psychometric properties of this instrument are appropriate in terms of reliability (Cronbach’s alpha) (cognitive anxiety: *α* = 0.87; self-confidence: *α* = 0.93; somatic anxiety: *α* = 0.90) (Rodríguez et al., 2017). In the present study, we obtained Cronbach’s alpha scores (*α* = 0.84).

### 2.3. Data analysis

The analysis was descriptive and performed with different statistical tests such as mean, standard deviation, and bivariate correlations. Reliability analysis and structural equation modeling (SEM) were performed to test the relationships established in the hypothesized model. The Joreskog test was chosen to analyze the covariance structure (Joreskog, 1970; Werts et al., 1973) of a Multiple Indicators Multiple Causes Model (MIMIC). The Joreskog test was used taking account the latent variable, who is defined as a composite of a set of measures (i.e., the measures produce the constructs, which is called as formative indicators, and structural equation systems, such as MIMIC, are best suited to address this situation). To accept or reject the proposed model, a set of suitable indices was taken into account (Hu and Bentler, 1999): TLI (Tucker-Lewis index), SRMR (standardized root mean square residual), and RMSEA (root mean square error of approximation). Thus, the appropriate indices are TLI values greater than 0.95; SRMR values less than 0.06; and RMSEA values less than 0.08. The analyses were carried out using SPSS software (version 26) and the R statistical analysis program (version 2015) and the analysis modules belonging to the “Lavaan” package.

## 3. Results

Table 1 shows the relationships between each of the dimensions of emotional intelligence: emotional attention (EA), emotional clarity (EC), and emotional regulation (ER) and their relationship with cognitive anxiety (CCA), somatic anxiety (SSA), and self-confidence (SCA). The correlations between the variables were positive, reflecting the reciprocity between the study variables (Table 2).

**Table 1:** Descriptive statistics on the characteristics of the sample.

	Females	Males	Total	Less than 25	Over 25
First course	18 (39.1)	68 (58.1)	86	86 (97.8%)	2 (2.2%)
Second course	16 (34.8)	23 (19.7)	39	38 (97.4)	1 (2.6%)
Third year	6 (13%)	14 (12%)	20	20 (100%)	0
Total	40	105	145		
Master’s degree	6 (13%)	12 (10.3)	18	4 (22.3%)	17 (77.7%)
Total	46	117	163		

**Table 2:** Bivariate correlation analysis.

	AEM	CE	RE	ACG	ATC	ASS
AEM		0.127	0.263**	0.209**	0.141	0.183*
CE			0.508**	0.61	0.210**	0.084
RE				0.049	0.157*	0.059
ACG					0.305**	0.234**
ATC						0.223**
ASS						

*p < 0.05; **p < 0.01.

**Figure 1:**
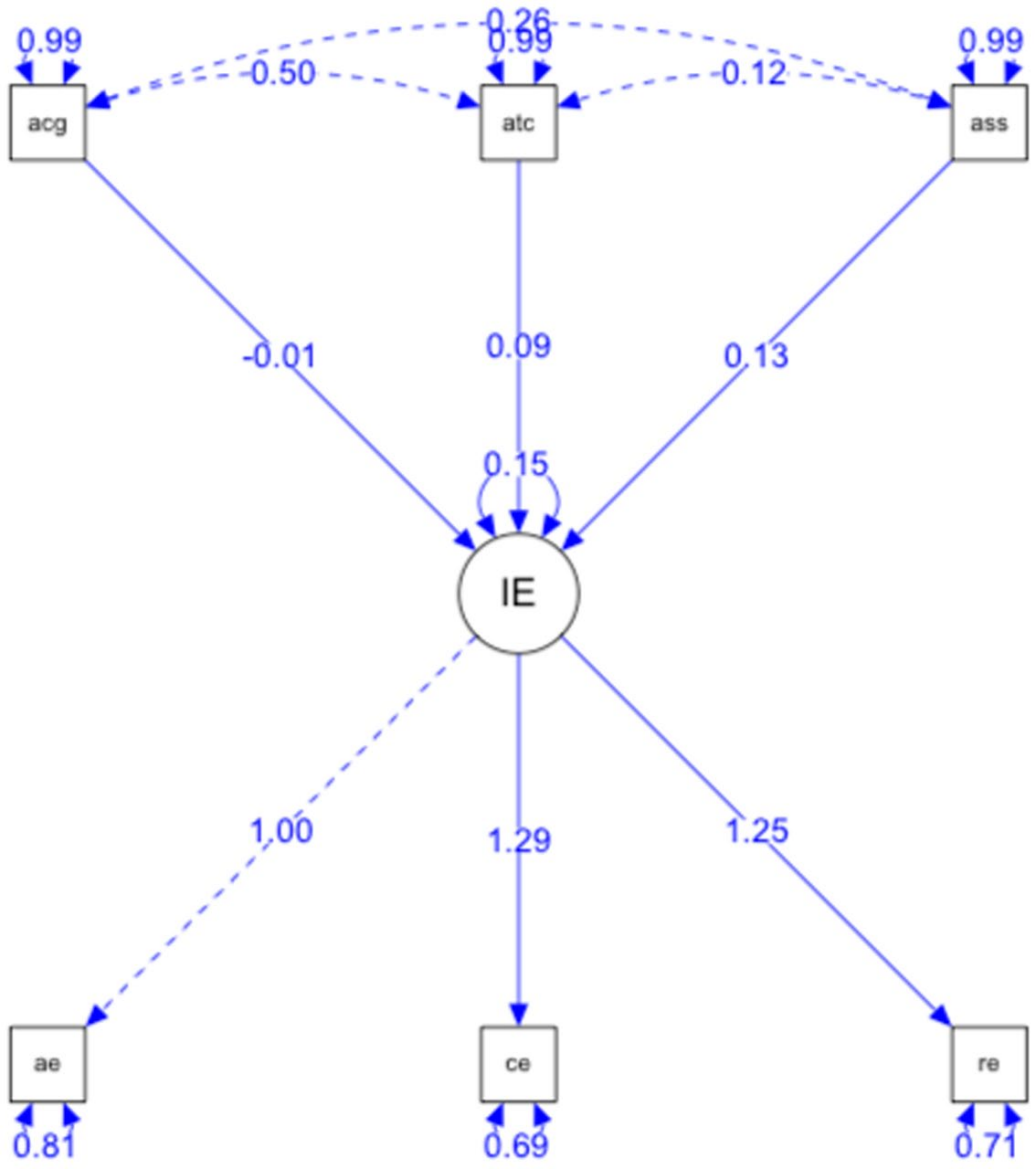
Structural equational modeling.

Testing the relationships established in the hypothetical model of predictive relationships (Figure 1), it has shown that the global fit indices (evaluating the model in general) were adequate: *p* < 0.001, RMSEA = 0.00, GFI = 0.982. As well as the incremental or comparative fit indices (comparing the proposed model with the model of independence or absence of relationship between the variables): NFI = 0,904; NNFI = 1,085; TLI = 0.963; CFI = 1; IFI = 1,036.

The parsimony indices (which assess the quality of the model fit in terms of the number of coefficients estimated to reach that level of fit) AGFI = 0.938.

In addition, two other indicators were calculated: Composite Reliability (CR), which indicates acceptable reliability (Heinzl et al., 2011), and the AVE (Average Variance Extracted) index, which measures the variance captured by a construct in relation to the other constructs in the model (Fornell and Larcker, 1981).

The results obtained through the SEM model in which the dimensions of emotional intelligence (attention, clarity, and regulation), and somatic anxiety, cognitive anxiety, and self-confidence are analyzed are as follows:

(a) Self-confidence and emotional intelligence correlated positively (=0.15, *p* < 0.001) which explains that, in this case, emotional intelligence is a predictor of self-confidence; therefore, the presence of this variable explains the existence of the other variable.

(b) Emotional attention and cognitive anxiety do not have a direct and positive relationship; therefore, the presence of this variable cancels out the existence of the other variable. In psychological terms, it can be considered a preventive factor.

(c) Emotional attention and somatic anxiety are not directly and positively related; therefore, the presence of this variable cancels out the existence of the other variable. In psychological terms, it can be considered a preventive factor.

## 4. Discussion

The aim of this study has been to analyze the relationship between anxiety and emotional intelligence in students related to physical activity and sport sciences. It should be clarified that in this case hypotheses 1 and 2 are fulfilled, while hypotheses 3 and 4 are not fulfilled. The main findings allow to affirm the relationship between emotional intelligence and anxiety, which demonstrate the fact that emotional intelligence corresponds to a series of skills related to the reflection of emotions and their viability to facilitate and govern thinking (Mayer and Salovey, 1997), which allows the subject to process, reason, and use emotions in a practical way in the face of various events in our daily lives (Salovey and Pizarro, 2003). The instrument used to measure anxiety enjoys a high prestige and validity, already used in research with large and diverse samples of different sportsmen and women, athletes, footballers, wrestlers, and surfers, among others (Caicedo Cavagnis et al., 2017; García et al., 2022).

In addition, within the sports field, it has been used for various purposes such as the relationship of anxiety with other psychological constructs such as endurance and heart rate and its effects on sports performance as well as with different types of psychological training such as progressive relaxation, etc. (Arruza et al., 2001; Sandoval and Armando, 2020; García et al., 2022; Mayorga et al., 2020; Teixeira and Remus, 2021).

It is important to highlight these results in terms of the relevance of the treatment of the emotional and psychological dimension in athletes and to stress their importance, together with it, the role of the sports psychologist. The results of the current study have shown that emotional intelligence can be a protective factor against negative feelings, emotions, or somatization, and therefore, we must implement it in sports practice, not only about physical or cognitive training, but also on an emotional level (Mayer and Salovey, 1997).

The results of this study are in the same line of other previous research that has aimed to estimate the psychological disposition of athletes and their sporting performance; emotions and bodily sensations in stressful situations have been studied (Ucha, 2004; Granero-Gallegos et al., 2011, 2017; López López, 2011; Fernandez-Berrocal et al., 2014). The main novelty and scientific advance of this study is the verification of the fact that emotional intelligence has a great positive impact on self-confidence, and therefore, by working on the emotional training of athletes we will also improve their self-confidence. The same happens with emotional attention, being demonstrated in this work that those athletes with high levels of emotional attention have less cognitive anxiety and less somatic anxiety. Therefore, emotional training is postulated here as a topic of great interest when it comes to minimizing anxiety and boosting self-confidence. This is also an educational implication from the training plans of future professionals in physical activity and sport sciences, so we advise that these aspects should be dealt with from their initial training.

Therefore, would be decisive to know the interpretation that each athlete makes of their emotional state so that high or low levels of anxiety could be treated for optimal performance in their competition. A possible surplus of anxiety would not necessarily be a negative aspect if it is helped to manage it, so in this sense, several studies have shown that elite athletes had lower levels of cognitive anxiety and higher levels of self-confidence than those of lower competitive level before and during the competition (León-Prados et al., 2011; Rodríguez Gómez and Granero Gallegos, 2017). Likewise, it has been found that higher level athletes recover earlier from their mistakes and have a higher level of self-confidence, so there is controversy regarding the establishment of a general profile of pre-competition anxiety necessary for optimal performance (López-Torres et al., 2007; Guerra Santiesteban et al., 2017; Garrido et al., 2018). These positions suggest that the perception of anxiety could be conditioned by the level of competition and by the characteristics of each sport discipline (Rodríguez Gómez and Granero Gallegos, 2017; Díaz, 2020). On the other hand, the importance of perceived self-confidence as a predictor of performance has been confirmed in other studies.

## 5. Limitations, practical implications, and future directions

Among the limitations of the study, we can point out that we have been cautious with the results provided by this study due to the size of the sample that made it up. Therefore, we believe that it would be productive to carry out this study again with a larger sample size in order to verify these findings, as this is the main limitation of this study. Another of the limitations encountered was the lack of theoretical background in terms of the subject matter, since the scientific contributions dealt with either sports anxiety or emotional intelligence, but not both together, as well as the fact that this is a cross-sectional study that does not measure the problem and its stability or change over time.

The practical implications of these results therefore involve the implementation of different actions or programs indicated to work on and improve emotional intelligence in athletes, based on the theoretical contributions of the ability models, which consider that emotional intelligence can not only be acquired, but also improved (Mayer and Salovey, 1997).

For future research, it would be advisable to inspect whether there are discrepancies according to the type of sport practiced and the sport practice. Thus, future research will try to probe discrepancies according to the degree of professionalization of each sport practiced by future participants. Therefore, we must highlight the need to continue to carry out research with future professionals in physical activity and sport sciences on these issues to give the importance that psychological training in emotions deserves, and not only the promotion of physical or cognitive training. It would also be interesting to design programs to work on, develop and raise awareness of emotional intelligence in the university world in general, and in degrees related to physical activity and sport in particular, and then, after a certain period of time, to analyze and evaluate the effectiveness of the used programs.

## 6. Conclusion

The present manuscript provides evidence that there is a positive correlation between dimensions of emotional intelligence (attention, clarity, and regulation) and somatic anxiety, cognitive anxiety, and self-efficacy. Our study concludes that the relationship between emotional intelligence and self-confidence is direct and positive. Conversely, there is a direct and negative relationship between high levels of emotional attention and low levels of cognitive anxiety and somatic anxiety.

## Data availability statement

The raw data supporting the conclusions of this article will be made available by the authors, without undue reservation.

## Ethics statement

The studies involving human participants were reviewed and approved by the Name of the Ethics Committee: Bioethics Commission of the University of Almería Approval code: UALBIO2022/012 Approval date: July 14, 2022. The patients/participants provided their written informed consent to participate in this study.

## Author contributions

IM-R, NG, SS, AM, and SB-C: conceptualization, methodology, investigation, formal analysis, data curation, and original draft preparation. IM-R, NG, and SB-C: review and editing. NG and SB-C: final review. All authors contributed to the article and approved the submitted version.

## Conflict of interest

The authors declare that the research was conducted in the absence of any commercial or financial relationships that could be construed as a potential conflict of interest.

The reviewer RT declared a shared affiliation with the authors IM-R and NG to the handling editor at the time of review.

## Publisher’s note

All claims expressed in this article are solely those of the authors and do not necessarily represent those of their affiliated organizations, or those of the publisher, the editors and the reviewers. Any product that may be evaluated in this article, or claim that may be made by its manufacturer, is not guaranteed or endorsed by the publisher.

## References

[ref1] Aguinaga Í. Herrero-Fernández D. Santamaría T. (2021). Protective factor of coping strategies and group cohesion on psychological well-being in situations of competitive anxiety in soccer players. CPD 21, 86–101. doi: 10.6018/cpd.414281

[ref2] Arruza G. González R. Palacios M. Arribas G. Cecchini E. (2012). Validation of the competitive state anxiety inventory 2 (CSAI-2 RE) through a web application. Revista Internacional de Medicina y Ciencias de la Actividad Física y el Deporte. 12, 539–556.

[ref3] Arruza J. Telletxea S. Azurza A. Amenabar B. Balague G. (2001). Relation among the mood states and the pre-competitive anxiety in snowboarders. in Póster Presented at XXXV International Congress of Applied Psychology, Singapore, Malaysia.

[ref4] Balk Y. A. de Jonge J. Oerlemans W. G. M. Geurts S. A. E. (2017). Testing the triple-match principle among Dutch elite athletes: a day-level study on sport demands, detachment and recovery. Psychol. Sport Exerc. 33, 7–17. doi: 10.1016/j.psychsport.2017.07.006

[ref5] Baro J. P. M. Garrido R. E. R. Hernández-Mendo A. (2016). Relaciones entre el perfil psicológico deportivo y la ansiedad competitiva en jugadores de balonmano playa. Rev. Psicol. Dep. 25, 121–128.

[ref6] Barwick G. S. C. Poyatos M. C. Fernández J. D. M. (2022). Inteligencia emocional y satisfacción con la vida en escolares durante tiempos de pandemia. Espiral. Cuadernos del Profesorado 15, 57–70. doi: 10.25115/ecp.v15i31.8209

[ref7] Botelho Vinhais J. Salselas V. (2013). Ansiedade pré-competitiva nas modalidades coletivas e individuais [Precompetitive anxiety in team sports and individual. Portugal. Available at: https://lume.ufrgs.br/handle/10183/248902

[ref9] Brito-Costa S.. (2022). Emotional intelligence of groups: psychometric properties of PIEGT16. Conf. Cephal. Neurol. 32:e2022002.

[ref10] Cabello R. Fernández-Berrocal P. Ruiz-Aranda D. Extremera N. (2006). Una aproximación a la integración de diferentes medidas de regulación emocional. Ansiedad y Estrés 12, 155–166.

[ref11] Cabello R. Ruiz-Aranda D. Fernández-Berrocal P. (2010). Docentes emocionalmente inteligentes. Rev. Elect.Interuniv. Formación Prof. 13, 41–49.

[ref12] Cahuasqui L. A. A. M. (2022). Ansiedad precompetitiva y su influencia en aspectos psicológicos del rendimiento deportivo en jugadoras profesionales de fútbol. Cienc. Lat. Rev Cient Mult. 6, 439–456.

[ref100] Caicedo Cavagnis E. Pereno G. L. De la Vega M. R. (2017). Adaptación del Inventario Revisado de Ansiedad Estado Competitiva - 2 a población deportiva argentina. Interdisciplinaria 34, 389–405.

[ref13] Capdevila L. (1997). “Metodología de Evaluación en Psicología del Deporte” in Psicología del Deporte. ed. En J. (Madrid: Síntesis)

[ref14] Castro-Sánchez M. Zurita-Ortega F. Ramírez-Granizo I. Ubago-Jiménez J. L. (2020). Relación entre la inteligencia emocional y los niveles de ansiedad en deportistas. J. Sport Health Res. 12, 42–53.

[ref15] Del Bosque R. A. (2019). Análisis y valoración de las relaciones entre el estado de ánimo y la ansiedad en relación al resultado en jóvenes futebolistas. Apunts Educ.Físic. Dep. 35:132.

[ref16] Del Bosque R. A. Moral García J. González Rodríguez O. Arruza Gabilondo J. (2022). Influencia del resultado en la ansiedad de futbolistas iniciados. Rev. Iber. C.a Act. 11, 15–30. doi: 10.24310/riccafd.2022.v11i2.14410

[ref17] Díaz O. J. V. (2020). Influencia de ansiedad precompetitiva y motivación al éxito deportivo de atletas en artes marciales. Rev. Univ. 1, 77–82.

[ref200] Extremera N. Fernández-Berrocal P. (2009). Test de Inteligencia Emocional de MayerSalovey Caruso. [Mayer Salovey Caruso Emotional Intelligence Test]. Madrid: TEA.

[ref18] Fernandes M. G. Vasconcelos-Raposo J. Fernandes H. M. (2012). Psychometric properties of the CSAI-2 in Brazilian athletes. Universidade Federal do Rio Grande do Sul Instituto de Filosofia e Ciências Humanas Revista 25, 679–687. doi: 10.1590/S0102-79722012000400007

[ref19] Fernández E. M. A. Río G. L. Fernández C. A. (2007). Propiedades psicométricas de la versión española del Inventario de Ansiedad Competitiva CSAI-2R en deportistas. Psicothema 19, 150–155. 17295997

[ref20] Fernández-Berrocal P. Extremera N. (2005). La inteligencia emocional y la educación de las emociones desde el Modelo de Mayer y Salovey. Rev. Inter. Form. Profes. 19, 63–93.

[ref21] Fernández-Berrocal P. Extremera N. (2008). A review of trait meta-mood research. Int. J. Psych. Res. 2, 39–67.

[ref22] Fernandez-Berrocal P. Extremera N. Ramos N. (2004). Validity and reliability of the Spanish modified version of the trait meta-mood scale. Psychol. Rep. 94, 751–755. doi: 10.2466/pr0.94.3.751-755, PMID: 15217021

[ref300] Fernández-Berrocal P. Extremera N. Lopes P. N. Ruiz-Aranda D. (2014). When to cooperate and when to compete: emotional intelligence in interpersonal decision-making. J. Res. Pers. 49, 21–24. doi: 10.1016/j.jrp.2013.12.005

[ref400] Fornell C. Larcker D. F. (1981). Evaluating Structural Equation Models with Unobservable Variables and Measurement Error. J. Mark. Res. 18, 39–50. doi: 10.2307/3151312

[ref23] Fornell C. Cha J. (1994). Partial least squares. Adv. Methods Mark. Res. 407, 52–78.

[ref24] Gaetano R. Paloma F. G. Gaetano A. (2015). Anxiety in the youth physical and sport activity. Mediterr. J. Soc. Sci. 6:227. doi: 10.5901/mjss.2015.v6n3s2p227

[ref25] García Del Castillo-López A. García Del Castillo J. A. Marzo J. C. (2013). La relevancia de la inteligencia emocional en la prevención del consumo de alcohol. Información Psicológica 104, 100–111.

[ref26] García K. A. R. Martínez P. G. Alanís A. D. J. G. (2022). Ansiedad estado y falta de confianza como predictores del rendimiento de equipos de fútbol feminil. Enseñanza e Investigación en Psicología 4, 32–42.

[ref27] Garrido R. Delgado-Giralt J. López-Cazorla R. Hernández-Mendo A. (2018). Perfil psicológico deportivo y ansiedad estado competitiva en triatletas. Ver. Psic. Dep. 27, 125–132.

[ref28] Gómez R. P. Sánchez J. C. J. Del Pilar Méndez-Sánchez M. Jaenes-Amarillo P. (2016). El poder explicativo de la ansiedad en los estados de ánimo de deportistas españoles. Retos 30, 207–210. doi: 10.47197/retos.v0i30.50259

[ref29] Górriz A. B. Etchezahar E. Pinilla-Rodríguez D. E. Giménez-Espert M. C. Soto-Rubio A. (2021). Validation of TMMS-24 in three Spanish-speaking countries: Argentina, Ecuador, and Spain. Environ. Res. Public Health 18:9753. doi: 10.3390/ijerph18189753, PMID: 34574687 PMC8469647

[ref30] Granero-Gallegos A. Gómez-López M. Rodríguez-Suárez N. Abraldes J. A. Alesi M. Bianco A. (2017). Importance of the motivational climate in goal, enjoyment, and the causes of success in handball players. Front. Psychol. 8:2081. doi: 10.3389/fpsyg.2017.02081, PMID: 29250011 PMC5717397

[ref31] Granero-Gallegos A. Lopez Gomez M. López Valeiras J. Suarez N. (2011). Motives of practice in the field of non-competitive physical activity. Espiral 4, 15–22. doi: 10.25115/ecp.v4i7.915

[ref32] Guerra Santiesteban J. R. Gutierrez Cruz M. Zavala M. Singre Álvarez J. Goosdenovich Campoverde D. Romero Frómeta E. (2017). Relación entre ansiedad y ejercicio físico. Rev. Cubana Inv. Bioméd. 36, 169–177.

[ref33] Guillén F. Álvarez-Malé M. L. (2010). Relación entre los motivos de la práctica deportiva y la ansiedad en jóvenes nadadores de competición. Rev. Iber. Psychol Exer. Dep. 5, 233–252.

[ref34] Haase M. (2021). Ansiedad precompetitiva y motivación en nadadores costarricenses de élite. MHSALUD Revista en Ciencias del Movimiento Humano y Salud 18, 1–18. doi: 10.15359/mhs.18-2.6

[ref35] Heinzl A. Buxmann P. Wendt O. Weitzel T. (Eds.) (2011). Theory-Guided Modeling and Empiricism in Information Systems Research. Heidelberg: Physica-Verlag.

[ref36] Hofrege S. Ruiz-Barquín R. Molinero O. (2018). Ansiedad y estrategias de afrontamiento en judokas de competición. Revista de Artes Marciales Asiáticas 13, 23–26. doi: 10.18002/rama.v13i2s.5501

[ref37] Hu L.-T. Bentler P. M. (1999). Cutoff criteria for fit indexes in covariance structure analysis: conventional criteria versus new alternatives. Struct. Equ. Model. Multidiscip. J. 6, 1–55. doi: 10.1080/10705519909540118

[ref38] Jara-Moreno Á. González-Hernández J. Gómez-López M. (2020). Perfeccionismo y ansiedad competitiva en jóvenes deportistas españoles. Anuario de Psicología 50, 57–65. doi: 10.1344/ANPSIC2020.50/2.31929

[ref39] Joreskog K. G. (1970). A general method for analysis of covariance structures. Biometrika 57, 239–251. doi: 10.1093/biomet/57.2.239

[ref40] Kumar A. (2016). A study of pre-competitive anxiety involving male and female players competing in team versus individual events. Int. J. Physic. Educ. Sports Health 3, 135–137.

[ref41] León-Prados J. A. Fuentes I. Calvo Á. (2011). Ansiedad estado y autoconfianza precompetitiva en gimnastas. (precompetitive anxiety state and self-confidence in gymnasts). Rev Int Cienc Deporte 7, 76–91. doi: 10.5232/ricyde2011.02301

[ref42] Liberal R. Tomàs Escudero J. Ponseti Verdaguer F. J. (2014). Impacto psicológico de las lesiones deportivas en relación al bienestar psicológico y la ansiedad asociada a deportes de competición. Rev. Psicol. Dep. 23, 451–456.

[ref43] López López I. S. (2011). *La evaluación de variables psicológicas relacionadas con el rendimiento en fútbol: habilidades psicológicas para competir y personalidad resistente*. Tesis Departamento de Educación Física y Deportiva. Univ, Granada.

[ref44] López M. G. Sánchez S. A. Ponce J. F. (2020). Factores de estrés y ansiedad en el arbitraje de deportes de equipo: una revisión sistemática. Espiral. Cuadernos del Profesorado 13, 74–85. doi: 10.25115/ecp.v13i26.2734

[ref45] López Walle J. M. Pineda Espejel H. A. Tomás I. (2014). Validación de la versión mexicana del CSAI-2R en sus escalas de intensidad y dirección. Rev. Mexic. Psicol. 31, 198–212. doi: 10.30552/ejihpe.v8i2.250

[ref46] López-Torres M. Torregrosa M. Roca J. (2007). Características del flow, ansiedad y estado emocional, en relación con el rendimiento de deportistas de élite. Cuadernos de Psicología del Deporte 7, 25–44.

[ref47] Marholz P. O. Contreras L. M. V. Barrera J. (2022). Niveles de Ansiedad Rasgo y Bienestar en jugadores de fútbol profesional de Chile durante la cuarentena por COVID-19. Retos 44, 1037–1044. doi: 10.47197/retos.v44i0.91316

[ref48] Martens R. Schwenkmezger P. (1979). Sport competition anxiety test. Sportwissenschaft 9, 101–103. doi: 10.1007/bf03177079

[ref49] Martens R. Vealey R. S. Burton D. (1990). Competitive Anxiety in Sport. Champaign, IL: Human Kinetics.

[ref50] Martens R. Ferrand C. Guillet E. Gautheur S. (2014). Reliability and validity of the competitive state anxiety inventory (CSAI). Motriz 20, 158–166. doi: 10.1590/S1980-65742014000200005

[ref51] Mayer J. D. Salovey P. (1997). “What is emotional intelligence?” in Emotional Development and Emotional Intelligence: Educational Implications. eds. Salovey P. Sluyter D. J. (New York: Basic Books), 3–34.

[ref52] Mayer J. D. Salovey P. Caruso D. R. (2008). Emotional intelligence: new ability or eclectic traits? Am. Psychol. 63, 503–517. doi: 10.1037/0003-066X.63.6.503 18793038

[ref53] Mayorga Lascano P. M. Jaramillo Á. Moreta-Herrera R. (2020). Ansiedad competitiva y autoeficacia en tenistas de alto rendimiento antes y después de una competência. Revista Guillermo de Ockham 18, 45–54. doi: 10.21500/22563202.4526

[ref54] Medeiros T. T. Santos R. W. Costa F. S. M. Santos R. W. (2021). Ansiedad precompetitiva en deportistas de judo tras suspensión de competiciones por las medidas restrictivas del COVID-19. Rev. Peru. Cienc. Act. Fís. Deporte 9:8. doi: 10.53820/rpcafd.v9i1.194

[ref55] Mellalieu S. D. Neil R. Hanton S. Fletcher D. (2009). Competition stress in sport performers: Stressors experienced in the competition environment. J. Sports Sci. 27, 729–744. doi: 10.1080/02640410902889834 19424897

[ref56] Morales-Beltrán R. A. Hernández-Cruz G. González-Fimbres R. A. Rangel-Colmenero B. R. Zazueta-Beltrán D. K. Reynoso-Sánchez L. F. (2022). La actividad física Como moderador en la ansiedad asociada al COVID-19 en estudiantes universitarios (physical activity as a moderator in anxiety associated to COVID-19 in university students). Retos 45, 796–806. doi: 10.47197/retos.v45i0.92974

[ref500] Moisao A. Brito-Costa S. Almeida H. Maldonado-Briegas J. Castro F. (2022). Burnout syndrome: symptoms, psychosocial variables and implications for sports. Confinia Cephagica et. Neurologica 32:e2022002.

[ref57] Pineda-Espejel H. A. Morquecho-Sánchez R. Alarcón E. (2019). Estilos interpersonales, factores disposicionales, autoconfianza y ansiedad precompetitiva en deportistas de alto rendimiento: Interpersonal styles, dispositional factors, self-confidence and precompetitive anxiety in high performance athletes. Cuadernos de Psicología del Deporte 20, 10–24. doi: 10.6018/cpd.397001

[ref58] Prieto J. M. Palmeira A. L. Olmedilla A. (2015). Ansiedad competitiva, competitividad y vulnerabilidad a la lesión deportiva: perfiles de riesgo. Rev. Iber. Psicol Exerc. Dep. 10, 293–300.

[ref59] Pulido S. Fuentes J. P. De la Vega R. (2021). Motivación, autoconfianza y ansiedad en judo: sexo y nivel competitivo. Int. J. Med. Sci. Phys. Act. Sport 21, 319–335. doi: 10.15366/rimcafd2021.82.008

[ref60] Ramis Y. Torregrosa M. Cruz J. (2013). Revisitando a Simon & Martens: La ansiedad competitiva en deportes de iniciación [Simon & Martens revisited: Competitive anxiety in youth sports]. Rev. Psic. Dep. 22, 77–83.

[ref61] Ramis Y. Torregrosa M. Viladrich C. Cruz J. (2010). Adaptación y validación de la versión española de la Escala de Ansiedad Competitiva SAS-2 para deportistas de iniciación [Adaptation and validation of the Spanish version of the Sport Anxiety Scale SAS-2 for young athletes]. Psicothema 22, 1004–1009. PMID: 21044545. PMID: 21044545

[ref62] Ries F. Castañeda Vázquez C. Campos Mesa M. C. Castillo Andrés O. D. (2012). Relaciones entre ansiedad-rasgo y ansiedad-estado en competiciones deportivas. Cuadernos de Psicología del Deporte 12, 9–16. doi: 10.4321/s1578-84232012000200002

[ref63] Rodríguez Gómez J. M. Granero Gallegos A. (2017). Mood, self-confidence and pre-competitive anxiety in sport shooting. Espiral Cuadernos del Profesorado 7, 13–23. doi: 10.25115/ecp.v7i14.967

[ref64] Rodríguez C. G. Sosa-Correa M. Zayas García A. Guil Bozal R. (2017). Regulación emocional en jóvenes deportistas ante situaciones adversas en competición. Revista INFAD de Psicología 2, 373–384. doi: 10.17060/ijodaep.2017.n1.v2.950

[ref65] Salovey P. Detweiler-Bedell B. T. Detweiler-Bedell J. B. Mayer J. D. (2008). Emotional Intelligence. Handbook of Emotions. New York: The Guilford Press.

[ref66] Salovey P. Pizarro D. A. (2003). “The value of emotional intelligence” in Models of Intelligence: International Perspectives. eds. Sternberg R. J. Lautrey J. Lubart T. I. (Washington, D.C: American Psychological Association), 263–278.

[ref67] Sandoval B. Armando E. (2020). Agilización en el procesamiento de datos y resultados en la evaluación diagnóstica de ansiedad precompetitiva del test CSAI-2R propuesto en tenistas de campo en etapa de especialización. Bogota, Colombia: Universidad Pedagógica Nacional.

[ref68] Sarudiansky M. (2013). Anxiety, anguish and neurosis. Influence Microgravity Repair Radiation Induced DNA Damage Bacteria Human Fibroblasts 21, 19–28. doi: 10.48102/pi.v21i2.151

[ref69] Sepúlveda-Páez G. Díaz-Karmelic Y. Ferrer-Urbina R. (2019). Pre-competitive anxiety and sports coping strategies, in individual and collective aquatic disciplines in high-level youth athletes. Límite 14, 1–10. doi: 10.4067/s0718-50652019000100216

[ref70] Silva D. Junior C. Junior J. Carvalho C. Gorla J. Araújo P. (2015). Differences between high-performance and base level handball players in motivational variables and pre-competitive anxiety. E-handball. Rev Cien. Deporte 11, 105–106. doi: 10.4025/jphyseduc.v32i1.3252

[ref71] Soper D.S. (2022). A-priori sample size calculator for structural equation models [software]. Available at: https://www.danielsoper.com/statcalc

[ref72] Spielberger C. D. Gorsuch R. L. Lushene R. (1970). Manual for the State-Trait Anxiety Inventory. Palo Alto, CA: Consulting Psychologists Press.

[ref73] Teixeira K. C. Remus J. B. (2021). Three-dimensional scale of anxiety for sport: development and evidence of validity based on non-content. Psico-USF 26, 241–251. doi: 10.1590/1413-82712021260204

[ref74] Telletxea S. (2008). *Application of a Psychosocial Intervention Program Aimed at Training and Optimization of Performance Capabilities in Athletes*. Doctoral thesis. Serie Tesis. Servicio Central de Publicaciones del Gobierno Vasco, Vitoria-Gasteiz

[ref75] Torrealva D. T. Bossio M. A. R. (2018). Program based on "mindfulness" for the reduction of pre-competitive anxiety in martial arts athletes. Retos 36, 418–426. doi: 10.47197/retos.v36i36.66589

[ref76] Ucha F. E. G. (2004). Herramientas Psicológicas para Entrenadores y Deportistas. La Habana: Editorial Deportes.

[ref77] Vaca García M. R. Egas R. S. R. García Q. O. F. Feriz O. L. Rodríguez T. Á. F. (2017). Pre-competitive anxiety in high-performance, amateur and novice karate fighters. Rev.Cub. Inv. Bioméd. 36, 239–247.

[ref78] Vivar-Bravo J. Pérez Y. Ynjante O. Núñez E. Ocaña-Fernández Y. (2022). Anger and anxiety in athletes from 18 to 39 years of age from metropolitan Lima and Callao during the covid 19 pandemic. Arch. Venez Farmac. Terap. 41, 34–38. doi: 10.5281/zenodo.6370328

[ref79] Weinberg R. Gould D. (2010). Fundamentals of sport psychology and physical exercise. Human Kinetics Publishers.

[ref80] Werts C. E. Joreskog K. G. Linn R. L. (1973). Identification and estimation in path analysis with unmeasured variables. Am. J. Sociol. 78, 1469–1484. doi: 10.1086/225474

[ref81] Zurita F. Moreno Arrebola R. González Valero G. Viciana Garófano V. Martínez Martínez A. Muros Molina J. J. (2018). Conceptual review of the connection between emotional intelligence and physical self-concept. SPORTK 7, 139–144. doi: 10.6018/322001

